# Independent Journalism

**Published:** 1880-02

**Authors:** 


INDEPENDENT JOURNALISM.—Already the course adopted in the inception of this enterprise is beginning to bear rich and valued fruit in the support and patronage it is receiving from enlightened and generous members of the profession generally, and from the commercial centres in which it has made its advent particularly. Verbal and written words of cheer and encouragement come to us from lips and pens unused to adulatory expressions; from men whom all respect and recognize as leaders in thought and work in the grand old art of healing. Could a doubt have lingered in the inauguration of this journal as to the necessity, or at least the propriety of its establishment, it would not now have a single peg to hang itself upon! Wise and good men appear and urge us onward in our self-appointed work, and assure us we have but to closely pursue the course marked out in our prospectus, to secure early and complete success. As our aim will be directed to the procurement of valuable information, and the discussion of matters of interest and importance to our readers, we shall

> "Seize the truth where'er 'tis found,
> Among our friends, among our foes,
> On Christian, or on heathen ground,
> The flower, divine where'er it grows."

It would be pleasant, and we feel quite strongly the temptation to do so, to place in enduring type the cheering words thus far received from generous friends, so that others in the future, as well as ourselves, may contrast and verify their prognostications and kindly expressed wishes for our success, with the accomplished fact. But our limited space must be occupied with more unselfish matter. We trust we may be pardoned for adding, however, that all that has been done for our encouragement and success will be warmly cherished by us long beyond the time of the *complete fulfillment* of our hopes and aspirations.

